# Enhancing Pharmaceutical Product Quality With a Comprehensive Corrective and Preventive Actions (CAPA) Framework: From Reactive to Proactive

**DOI:** 10.7759/cureus.69762

**Published:** 2024-09-19

**Authors:** Thirumalai Arunagiri, Kanaka P Kannaiah, Manimaran Vasanthan

**Affiliations:** 1 Department of Pharmaceutical Quality Assurance, Sri Ramaswamy Memorial (SRM) College of Pharmacy, Sri Ramaswamy Memorial (SRM) Institute of Science and Technology, Chennai, IND

**Keywords:** 8d methodology, capa, corrective action, preventive action, root cause analysis

## Abstract

Corrective and preventive actions (CAPA) are crucial components of quality assurance (QA) within the pharmaceutical industry, essential for maintaining product quality, safety, and regulatory compliance. The review explores the multifaceted role of CAPA in pharmaceutical manufacturing, emphasizing its structured approach to detecting, addressing, and preventing quality issues. CAPA systems are integral to the broader quality management system (QMS), functioning as a dual-loop mechanism that is reactive and proactive approach aligned with continuous improvement principles outlined by the International Organization for Standardization (ISO) 9001:2000. It details the three distinct phases of CAPA: correction or remedial action, corrective action (CA), and preventive action (PA). It highlights the importance of root cause analysis and the necessity for immediate corrections and long-term preventive measures to avoid recurring issues. Regulatory expectations, such as those from the Food and Drug Administration (FDA) under the Code of Federal Regulations (CFR) title 21 part 820 and the International Council for Harmonisation of Technical Requirements for Pharmaceuticals for Human Use (ICH) Q10, are discussed, underscoring the need for a comprehensive CAPA plan that integrates data analysis and ongoing process enhancements. Additionally, the paper introduces the 8D methodology as a structured problem-solving approach to complement CAPA efforts. By providing an in-depth examination of CAPA procedures and their implementation, this article aims to contribute to the understanding and effectiveness of quality systems in pharmaceutical manufacturing.

## Introduction and background

Corrective and preventive actions (CAPA) are vital for ensuring quality assurance (QA) within the pharmaceutical industry. The CAPA system is structured to detect, address, and avert issues concerning product quality, safety, and regulatory compliance. Implementing an effective CAPA system is crucial for upholding the rigorous standards of drug manufacturing and ensuring that all products meet required safety and efficacy benchmarks. When a flaw is identified, whether related to production or testing, an investigation into the root causes must commence immediately [1]. Corrective actions (CA) should be taken to resolve the current nonconformity or quality issue, while preventive actions (PA) aim to mitigate the recurrence of similar problems. Nonconformity refers to the failure to meet a specific requirement. When a nonconformance or an undesirable condition occurs, the initial response is to take corrective or remedial action. This action focuses on addressing the immediate issue, providing a temporary solution that resolves the current problem without tackling the root cause. In contrast, CA involves a comprehensive approach to eliminate the root cause of the nonconformity. This process not only addresses the identified issue and contains it through immediate measures but also ensures that the problem does not recur. According to earlier versions of the International Organization for Standardization (ISO) 9001, corrective actions are designed to prevent the issue from spreading and recurring [2]. PA differs by targeting potential problems before they occur. It involves identifying possible process issues, determining their causes, and implementing measures to eliminate these causes, thereby preventing nonconformities in the future [3]. The process of CA and PA involves several steps: identifying the likely source of the potential problem or nonconformance, determining the cause, developing a preventive plan, implementing it, and finally evaluating the effectiveness of the actions taken in preventing the issue [4]. The main goal of CAPA in the pharmaceutical or medical device industry is to identify weaknesses, deviations, or failures and to investigate them thoroughly, implementing appropriate actions to ensure these issues do not reoccur. The Food and Drug Administration (FDA) often criticizes addressing only the immediate problem as a "band-aid approach", which frequently leads to warning letters. CAPA is an integral component of the broader quality management system (QMS) [5].

CAPA embodies the continuous improvement philosophy outlined by the International Organization for Standardization (ISO) 9001:2000. As a continuous and robust element of product, process, and quality system enhancement, CAPA functions as a quality improvement tool operating on a dual-loop system that is one reactive and one proactive. The FDA's updated systemic audit inspection approach, which emphasizes risk-based management, underscores the necessity for a proactive CAPA program [6].

In many instances, the root cause of an issue reflects deeper organizational or market-related flaws, and retraining alone is insufficient to conclude a CAPA. CA is one of the most critical improvement activities within a CAPA system. It involves identifying the underlying causes of a problem and implementing measures to eliminate these causes, thereby preventing recurrence. On the other hand, PAs are designed to preclude potential issues before they arise [7].

Quality systems in the pharmaceutical industry are governed by the FDA under the Code of Federal Regulations (CFR) title 21 part 820, known as the "quality system regulation" (QSR). To assist FDA inspectors in evaluating compliance with QSR requirements, the FDA developed the Quality Systems Inspection Technique (QSIT). QSIT concentrates on four key subsystems: management controls, design controls, CAPA, production, and process controls are considered the foundation of a quality system. The other three subsystems facilities, and equipment controls, materials controls, and document/record/change controls, are examined in conjunction with the primary four [8]. The 8D methodology employs eight disciplines or principles for problem-solving. Its objective is to effectively identify and define the problem statement, enabling necessary CAPA to prevent the recurrence and occurrence of issues [9]. CAPA encompasses three distinct phases like correction or remedial action, CA, and PA, each vital for systematically addressing and preventing quality issues, thereby ensuring continuous improvement in pharmaceutical manufacturing.

A typical example of CAPA in the pharmaceutical industry is the investigation and resolution of a batch failure. Suppose a pharmaceutical company identifies that a batch of products has failed quality testing due to a packaging defect. In response, the company takes CA by quarantining and recalling the failed batch, investigating the root cause of the packaging defect, and resolving the issue. To prevent recurrence, the company implements PA by reviewing and updating packaging procedures, providing additional training for packaging personnel, and enhancing quality checks [10]. The difference between CA and PA is tabulated in Table 1.

**Table 1:** Difference between CA and PA CA - corrective action; PA - preventive action

Corrective action	Preventative action
Action was taken on the root cause to prevent recurrence.	Action taken on potential causes of failures to prevent occurrence.
Reactive approach	Proactive approach
Deals with a nonconformity that has occurred.	Deals with potential nonconformity no failure has yet occurred.
Focus on past events	Focuses on potential future events
Requires root cause analysis	Requires risk or trend analysis
Documentation for a corrective action serves as proof that the issue was identified, resolved, and that appropriate controls were implemented to prevent its recurrence.	Documentation for a preventive action demonstrates that an effective quality system is in place, capable of anticipating, identifying, and eliminating potential issues before they arise.

Purpose of the CAPA subsystem

The objective is to identify recurring quality issues by utilizing appropriate statistical methods to gather and analyze relevant data. This process enables the implementation of effective, targeted CAPA. Additionally, it involves identifying and investigating both existing and potential product and quality concerns, ensuring a comprehensive approach to maintaining and improving product standards [11].

## Review

Expectations from regulatory bodies: ICH Q10

Pharmaceutical and medical device companies must establish a CAPA system that is guided by the evaluation of complaints, product rejections, non-conformances, recalls, deviations, audits, regulatory inspections, as well as trends in process performance and product quality [12]. It is essential to use a structured investigative approach to pinpoint root causes, ensuring that the effort, formality, and documentation are proportionate to the risk level, as specified in ICH Q9 (Quality Risk Management). The CAPA process should result in better product and process insights, leading to overall quality improvements. This approach should not only refine processes and products but also enhance their understanding of them (Code of Federal Regulations 2015) [13].

21 CFR 820 regulatory requirements (procedures)

Manufacturers are required to develop and maintain a comprehensive plan for implementing CAPA. This plan must include protocols that ensure the CAPA subsystem requirements are fully met. The organization is responsible for defining and documenting, whether electronically or physically, and executing the plan. The complexity and scope of these procedures may vary depending on the organization's size and specific needs, allowing for a tailored approach to CAPA implementation [4].

CAPA data analysis

CAPA process entails examining work activities, responsibilities, audit reports, quality standards, service records, complaints, returned products, and various other quality data sources to pinpoint the actual and potential causes of nonconforming products or other quality issues. To detect recurring quality problems, suitable statistical methods will be utilized as needed [4].

CAPA subsystem

**Figure 1:**
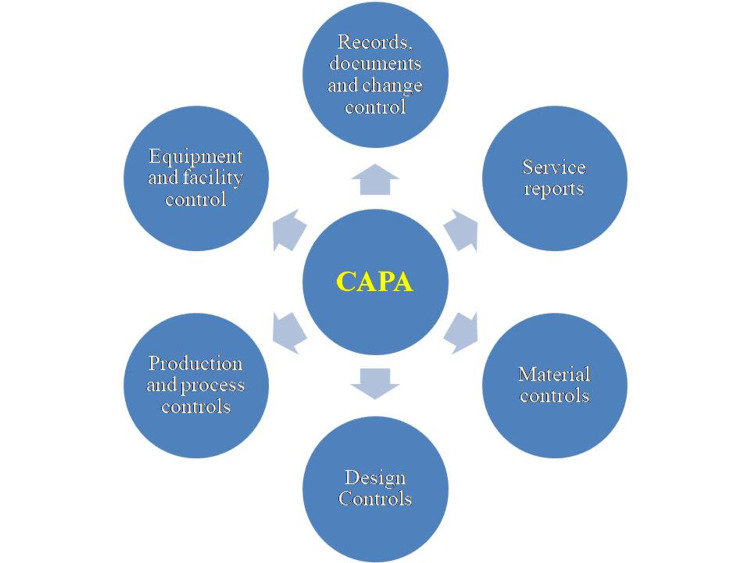
Subsystems of CAPA CAPA - corrective and preventive action Figure created by authors.

The CAPA system is essential for an effective QMS and must be closely integrated with other quality subsystems which are depicted in Figure 1. The primary goal for any regulated company is to ensure that its CAPA system is compliant, efficient, and effective. This system should involve all relevant subsystems that could generate non-conformances, including internal processes like non-conformance results, audits, assessments, and management reviews, as well as external sources such as supplier audits, customer feedback, and regulatory assessments.

To identify the causes of quality issues, both internal and external data sources should be utilized. Internal sources include inspection data, process control data, equipment maintenance records, and non-conformance reports, while external sources may include customer complaints, field service reports, and feedback from regulatory bodies. When a quality problem is detected, a failure investigation must be conducted to identify its root cause, ensuring that procedures were followed and controls were sufficient to prevent defective products from reaching the market. The depth of the investigation should align with the severity and risk of the issue. Appropriate corrective and preventive actions should be determined to address the problem and prevent its recurrence, with verification to ensure these actions are effective and do not negatively impact the products [8].

Process of CAPA

There are seven steps involved in CAPA in the pharmaceutical industry.

Identification of the Issue

The first step in CAPA process is to precisely define the problem [8]. Identify the source of information; examples of sources that might trigger a PA include service requests, internal quality audits, customer complaints or concerns, quality assurance inspections, staff observations, risk assessments, process performance monitoring, management reviews, and failure mode analysis. To effectively address an issue, begin with a clear and concise description that thoroughly outlines the problem. It is crucial to document the evidence supporting the problem's existence, as this information is critical for investigation and action planning. Proper documentation also facilitates the evaluation of the quality system's effectiveness and ensures that the resolution is clearly communicated to the relevant parties [6].

Risk Assessment

Once an issue is documented, it should be evaluated to decide if and what level of action is needed. This involves assessing the impact and risks to check potential threats to the business and its clients. The evaluation should detail the issue's significance, including possible effects on costs, product quality, safety, or reliability. The findings from this impact analysis will help to determine the severity of the issue [6].

Development of an investigation plan

After evaluating the potential impact and risks of the situation, it is essential to develop a procedure for investigating the problem. The written plan should include key elements to ensure a comprehensive investigation: the purpose of the actions to be taken, the investigative methods and timelines, and the assigned responsibilities and required resources [14].The newly developed investigation process should be used to explore the root cause of the problem. This involves collecting relevant data, examining all possible sources of the issue, and analyzing the information to pinpoint the primary cause. The manufacturer should create a comprehensive list of potential causes, which will guide the collection of data, test results, and other relevant information. It is crucial to organize and document the findings, including test outcomes and reviews of documents and procedures. This documentation should address all identified potential causes, as it will be essential in determining the root cause of the problem [5].

Root Cause Analysis (RCA) and Methods

Determining the root cause involves asking a series of "why" questions to dig deep until the underlying issue are identified. Effective root cause analysis is crucial for successful CA, as without it, corrective measures may be ineffective [15].

Statistical methods include tools such as Statistical Process Control (SPC), which utilizes Shewhart and Pareto charts, as well as regression analysis, encompassing both linear and non-linear forms. Additionally, the design of experiments (DOE) is employed for variance studies and experimental modeling. Pictographic techniques, like circle graphs and scatter diagrams, are also part of these methods. On the other hand, non-statistical methods comprise administrative reviews, which involve evaluating processes and conducting quality discussions. Failure mode and effects analysis (FMEA) is another key method for assessing potential failure modes and their impact on product performance. The fault tree analysis, a deductive approach, is used to graphically represent the causes of failures, particularly in the analysis of complaints or deviations [16].

**Figure 2:**
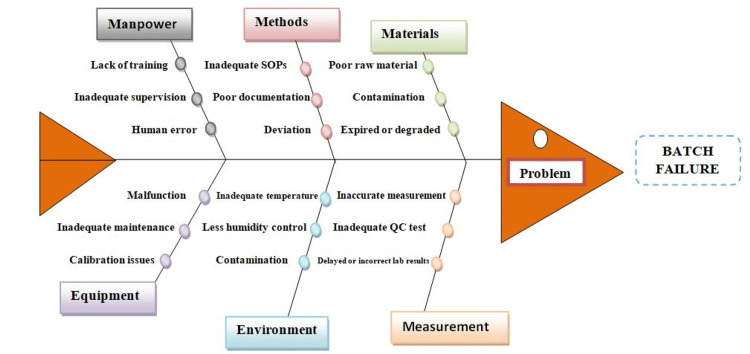
Fishbone analysis illustrating the identification, cause and analysis of factors contributing to batch failures in the pharmaceutical industry SOP - standard operating procedure; QC - quality control Figure created by authors.

Fishbone analysis (Ishikawa diagram): The Ishikawa diagram identifies potential causes of quality issues by mapping out the relationship between effects and their causes. It promotes a systematic approach to problem-solving, encouraging group involvement and aiding in the identification of data for further analysis. The diagram classifies variables based on their potential impact and helps establish a control strategy. For example, in the pharmaceutical industry, this method can be particularly effective in analyzing batch failures as depicted in Figure 2, where it can pinpoint specific factors that may have contributed to the problem [17].

Problem analysis form: A problem analysis form can be used to document the study's details, serving as a central point for collecting and attaching relevant information, though its use is optional.

Formulation of an action plan

The findings of the study should be used to develop a CAPA action plan aimed at resolving and/or preventing the issue. This plan should outline the tasks to be completed, specify any necessary revisions to documents, specifications, and processes, and assign responsibilities to specific employees. It should also include staff training requirements and an estimated completion date. The manufacturer should list all documents that will be updated, along with a general description of the expected changes. Additionally, the plan should detail the anticipated outcomes of any modifications to processes, procedures, or systems, providing clear guidance on what needs to be accomplished [4].

Implementation and documentation

Once the CAPA action plan is established, it is essential to promptly start, complete, and document all identified tasks. Accurate documentation is critical for executing the plan effectively. An implementation summary should be created, detailing all actions taken as per the plan, including adjustments, preventive measures, process controls, and training. This summary provides a complete record of the steps undertaken to address the issue and prevent its recurrence. Any changes must be communicated to the relevant individuals and departments, and all revised documents and requirements should be thoroughly documented [14].

Review effectiveness

After implementing the CAPA plan, a thorough follow-up analysis is essential. The main goals are to verify that all tasks have been completed and to confirm that changes, controls, and training have been effectively implemented. This involves documenting the completion of these actions and evaluating their effectiveness. The evaluation should ensure that the root cause of the problem has been addressed, any secondary issues have been resolved, appropriate controls are in place, sufficient monitoring is established, and any negative effects of the actions have been managed [14].

Finalization and validation of CAPA

Upon completion of CAPA actions, the department head will certify that the CAPA and associated actions have been fully implemented. Quality assurance (QA) will then verify this implementation by reviewing the supporting documents and providing certification. Any changes resulting from CAPA must follow the standard operating procedure (SOP) for change control and be noted in the CAPA documentation. All issues leading to CAPA, such as change control, deviations, incidents, and major quality system changes, must be documented through the CAPA form. Records of each CAPA will be maintained, and a copy provided to the relevant department head by QA. QA will compile CAPA information and present a summary during management review meetings, which will be reviewed quarterly by management. Information and documents related to CAPA from audits or inspections are confidential and will only be accessible for regulatory review with approval from the Director of Technical and the QA head [6][18].

CAPA management system

**Figure 3:**
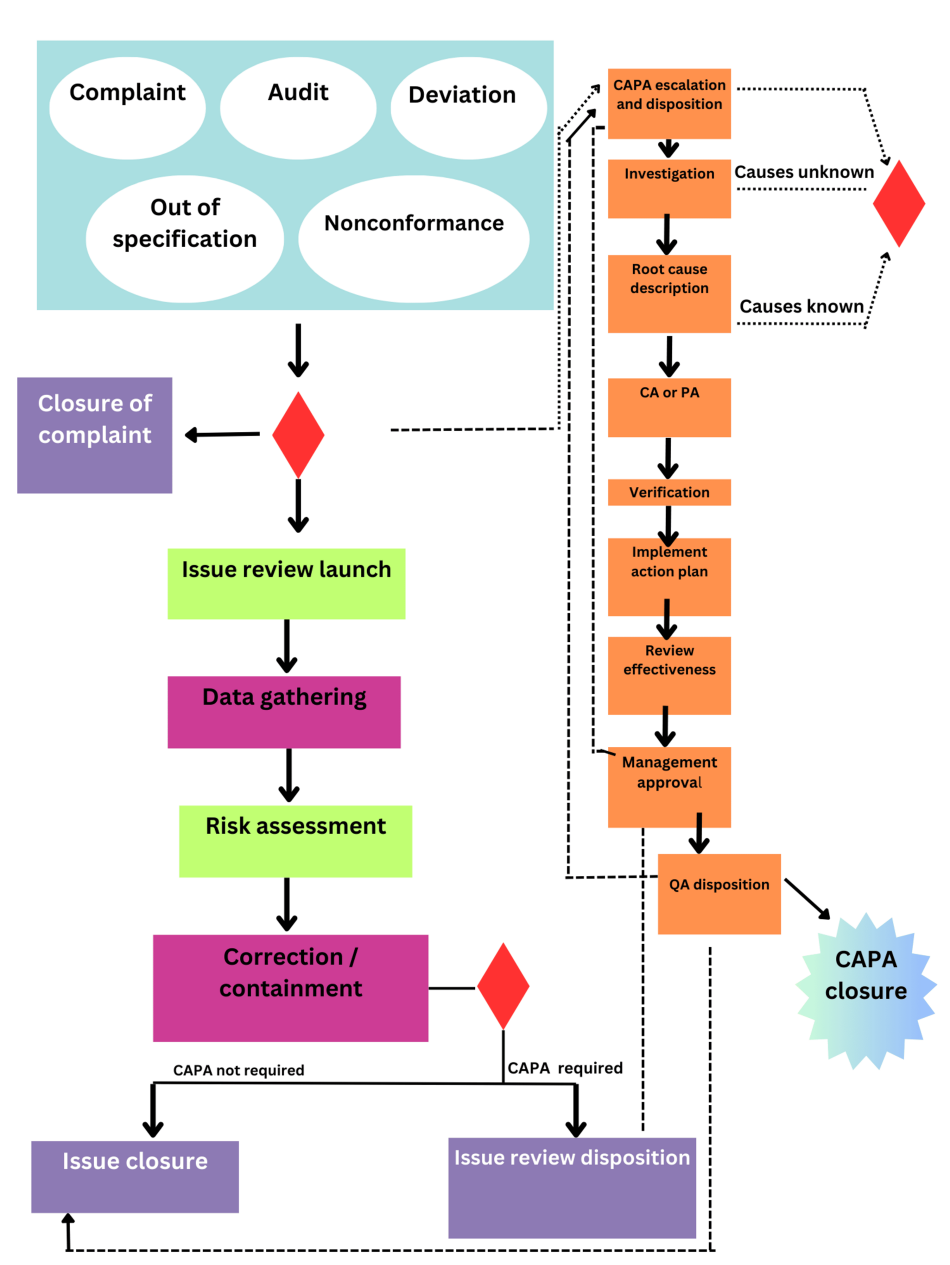
Flowchart of the CAPA management system CAPA - corrective and preventive action; CA - corrective action; PA - preventive action; QA - quality assurance Figure created by authors.

A CAPA management system is a critical component of a QMS in the medical technology industry [19]. It involves structured processes for identifying, documenting, and addressing both existing issues and potential risks. The system ensures that CA are taken to resolve identified problems, preventing their recurrence, while preventive actions are implemented to mitigate the risk of future issues. A well-designed CAPA management system includes thorough RCA, detailed action planning, and ongoing monitoring to verify the effectiveness of the actions taken. Proper documentation, regular management reviews, and a commitment to continuous improvement are essential to maintaining the system's effectiveness and ensuring compliance with industry standards and regulatory requirements is well illustrated in Figure 3 [5].

8D approach for problem resolution

The 8D methodology is a structured approach designed to address and resolve problems. Its main objectives are to pinpoint the root cause, implement corrective measures, and eliminate issues through a collaborative team effort, ultimately contributing to product and process enhancement. The key difference between CAPA is to provide a broad framework for addressing and preventing issues across various processes, focusing on continuous improvement. 8D offers a structured, step-by-step approach to problem-solving, specifically useful for detailed root cause analysis and resolution in manufacturing. The 8D methodology consists of eight crucial steps necessary to accomplish this goal.

Assembling of a Knowledgeable Team

To form the team, appoint a leader and select members with the necessary expertise related to the issue at hand. Ensure the team is cross-functional, comprising individuals with a thorough understanding of the product or process involved. Clearly define the team's goals and objectives to align efforts and guide the problem-solving process effectively.

Clear Definition of the Problem Using the 5W2H Method

The problem should be clearly defined with the issue using the 5W2H (what, when, where, who, which, how) method and a process flow diagram (PFD) and ensure the problem definition is based on facts, not opinions.

Implementation of an Immediate Containment Action

Containment actions, also known as interim or short-term actions, are immediate measures taken to address a problem and prevent defective or suspect materials from reaching the customer. These actions protect the customer's production line from quality issues while the root cause is investigated and countermeasures are developed. Examples include issuing quality alert notes, providing training on customer complaints, and segregating defective materials at various stages, such as work-in-progress, storage, dispatch, supplier, transit, and customer sites.

Identification and Confirmation of the Root Causes

RCA is a systematic method for pinpointing the underlying cause of a problem using tools like the five whys (5W) and Fishbone diagrams. To identify the root cause, start by brainstorming and utilizing a Fishbone diagram to explore all possible causes. Validate and select the most likely cause, then use the 5W technique to drill down to the root issue. Ensure the root cause addresses both occurrence and detection aspects and verify its accuracy. Avoid attributing the root cause solely to factors such as operator negligence, which often points to gaps in the system or procedures.

Development and Validation of Long-Term Solutions

Identify and select permanent corrective actions (PCAs) that effectively address and rectify the root cause of the problem. These actions should resolve the issue for the customer. Choose the best solution from all available alternatives, and ensure to document and verify the PCA to confirm its effectiveness.

Implementation and Verification of Corrective Actions

Implement the most effective PCA and monitor them closely to ensure they work as intended. check for any negative side effects of these actions. If problems persist, revisit the root cause analysis to refine the actions or re-evaluate the problem to uncover a new root cause.

Adjustment of Systems to Prevent Recurrence

Ensure that CA are applied across similar machines, products, and services to prevent recurring issues. Update systems, processes, procedures, and documents like control plans, and FMEA to guard against recurrence. Implement Poka-Yoke techniques to enhance system and process reliability by preventing errors. Continuously monitor the effectiveness of these permanent CA to ensure they remain effective.

Recognition of the team's successful efforts

Congratulate your team on their successful problem-solving effort and celebrate the achievement. The organization should express gratitude to the team for their contributions. Document the lessons learned and display the lesson learned card (LLC) in relevant areas to share the insights gained [20,21].

## Conclusions

The CAPA system is an indispensable component of QA in the pharmaceutical industry, essential for maintaining high standards of drug manufacturing for ensuring quality and regulatory compliance. Systematically addressing both immediate and potential issues through a dual-loop approach like CA and PA promotes continuous improvement and operational excellence. The thorough investigation of non-conformities and the implementation of targeted actions are crucial for resolving existing quality issues and preventing their recurrence. The integration of CAPA within the QMS ensures that organizations not only comply with regulatory requirements but also enhance their processes and products. Regulatory frameworks, such as FDA's 21 CFR Part 820 and ICH Q10, mandate a structured CAPA approach that emphasizes thorough RCA, effective action planning, and ongoing monitoring. The 8D methodology further enriches the CAPA process by providing a systematic, team-oriented approach to problem-solving, ensuring that issues are addressed comprehensively and solutions are validated for long-term effectiveness. The successful application of CAPA requires a robust management system that encompasses detailed documentation, rigorous evaluation, and continuous refinement. In conclusion, the CAPA system, when effectively implemented, not only resolves current quality issues but also strengthens the organization's ability to anticipate and mitigate potential risks, fostering a culture of continuous improvement and regulatory compliance. The systematic approach to CAPA ensures that pharmaceutical companies uphold the highest standards of quality and safety, ultimately benefiting both the industry and its customers.
